# The Role of Inhibition in Age-related Off-Topic Verbosity: Not Access but Deletion and Restraint Functions

**DOI:** 10.3389/fpsyg.2016.00544

**Published:** 2016-04-26

**Authors:** Shufei Yin, Huamao Peng

**Affiliations:** ^1^Institute of Developmental Psychology, Beijing Normal UniversityBeijing, China; ^2^Department of Psychology, Faculty of Education, Hubei UniversityWuhan, China

**Keywords:** age-related off-topic verbosity, inhibition, access, deletion, restraint

## Abstract

The speech of older adults is commonly described as verbose and off-topic, which is thought to influence their social communication. This study investigated the role of inhibition in age-related off-topic verbosity (OTV). Inhibition consists of three functions: access, deletion, and restraint. The access function is responsible for preventing irrelevant information from accessing the attention center (pre-mechanism of inhibition); The deletion function is responsible for deleting previously relevant but currently irrelevant information from working memory, and the restraint function is responsible for restraining strong but inappropriate responses (post-mechanisms of inhibition). A referential communication task was used to determine whether OTV was influenced by the pre-mechanism of inhibition. A self-involved event interview task was used to investigate the effect of the post-mechanisms of inhibition on OTV. Results showed that the OTV of the elderly participants was associated with an age-related decline in the post-mechanisms of inhibition, while the OTV exhibited by young adults was most likely due to deficits in the pre-mechanism function of inhibition. This research contributed to fill gaps in the existing knowledge about the potential relationship between specific functions of inhibition and age-related OTV.

## Introduction

Off-topic verbosity (OTV) (Glosser and Deser, 1992; Arbuckle and Gold, 1993) entails an extended series of loosely connected recollections that become increasingly unrelated to the concept of the original stimulus, and thus attenuate sequential coherence. It is characteristic of the speech production of a minority of elderly individuals. The hallmark of OTV, in contrast to talkativeness, is the failure to maintain focus and coherence. The influence of age on OTV incidence has been studied extensively and the results consistently have found that elderly adults more frequently exhibit OTV compared with young adults (Cooper, 1990; Arbuckle et al., 2000; Beaudreau et al., 2005; Juncos-Rabadán et al., 2005; Horton and Spieler, 2007; Trunk and Abrams, 2009; Abrams and Farrell, 2011; Wright et al., 2011; Jensen, 2012; Wills et al., 2012).

An explanation for the age-related association in OTV was posed by inhibition deficit theory, proposed by Hasher and Zacks (1988). It is assumed that OTV was related to *inhibition*, which as a psychological process that essentially prevents irrelevant information from interfering with the representation of relevant information. Inhibition consists of three functions: access, deletion, and restraint. The access function is responsible for preventing irrelevant information from accessing the attention center and it is also called a pre-mechanism of inhibition. The deletion function is responsible for deleting previously relevant but currently irrelevant information from working memory, and the restraint function is responsible for restraining strong but inappropriate responses. The deletion and restraint functions mainly exert their effects after interfering information is activated and these functions can be referred to as post-mechanisms of inhibition (Hasher et al., 1991, 1999; Zacks and Hasher, 1997). In the elderly, random and irrelevant thoughts and topics are more likely to intrude (Gold and Arbuckle, 1995). Deficits of inhibition cause elderly people to talk about topics that are irrelevant to the actual topic and affect the entire speech production of elderly adults.

The inhibition ability gradually declines with age, and the age-related decline in this type of ability influences elderly people’s working memory skills, including the prefrontal cortical functions of launching, control and integrated goal-directed behavior (Kaye et al., 1990); these functions, in turn, affect the wider range of cognitive functions, including selective attention, episodic memory, language, etc. (Gernsbacher and Faust, 1991; Hasher et al., 1991; Murphy et al., 1999, 2006; Engelhardt et al., 2010; Cansino et al., 2011). Age-related OTV also results from age-related declines in the ability to inhibit irrelevant information from reaching working memory; i.e., in the elderly, random irrelevant thoughts and topics are more likely to intrude (Arbuckle and Gold, 1993; Gold and Arbuckle, 1995). Deficits of inhibition might cause elderly people to talk about topics that are irrelevant to the target topic and affect the entire language production of elderly adults (including production related to autobiographical and non-autobiographical topics).

The inhibition deficit hypothesis has received much support from empirical research (Glosser and Deser, 1992; Arbuckle and Gold, 1993; Arbuckle et al., 2000, 2004). Pushkar et al. (2000), who adopted the Controlled Oral Word Association Test (Benton et al., 1994) and the Stroop Neuropsychological Screening Test (Lezak, 1995) to measure inhibition function, found that higher OTV participants had lower cognitive inhibitory scores. Arbuckle and Gold (1993), who interviewed elderly people about their life events, found that levels of OTV, while discussing an autobiographical topic, were significantly related to measurements of the ability to inhibit irrelevant information from reaching working memory, rather than that of language and visual memory. Their measures included the Wisconsin Card Sorting Test (WCST) and the Trail-Making Test (TMT). In the WCST, participants were asked to discover the “correct” basis for classifying stimuli that differed on three dimensions: shape, color, and number. They were required to respond appropriately to shifts in sorting criteria, rather than persevere on previously correct responses. This test measures an individual’s ability to clear previous sorting criteria and obtain new sorting criteria; hence, it can serve as a possible measure of the deletion function of inhibition. The TMT measures the time taken by subjects to link randomly arranged letters (Form A), or randomly arranged letters and numbers (Form B), to an alphabetic or alphanumeric sequence. To perform the alphanumeric alternation on Form B, subjects must inhibit the strong sequential associations within the alphabetic and numeric sets, hence, it can serve as a measure of the restraint function of inhibition. The results of Arbuckle and Gold (1993) showed that verbosity was significantly related to performance on the TMT rather than performance on the WCST. This suggests that the restraint function rather than the deletion function might be correlated with OTV. However, no studies have been conducted to investigate directly, the role of the deletion and restraint functions on OTV. We also questioned the influence of the access function on OTV and decided to investigate it.

Previous studies (Arbuckle and Gold, 1993; Pushkar et al., 2000) have confined their research of OTV to older adults, and lower inhibition function is associated with higher OTV in elderly adults. The relationship between individual differences in inhibition function and OTV may reflect the age-related decline of a mechanism involved in OTV, which would explain why OTV is greater in older adults than in young adults. Thus, it was necessary to compare age differences in OTV between older adults and younger adults. The inhibition function of older adults was found to be poorer than that of younger adults (Dumas and Hartman, 2008; Feyereisen and Charlot, 2008), and through previous studies of individual differences in OTV, we inferred that the age-related declines in the inhibition function could explain age-related increases in OTV to a certain extent. However, to investigate further the relationship between age-related OTV and inhibition, the characteristics of the relationships between OTV and inhibition across different age groups needed to be compared.

Although the inhibition deficit theory can explain OTV in relation to general topics (including autobiographical and non-autobiographical topics), this theory and its supportive evidence has not provided a clear interpretation of the effects of age on OTV. The aim of the present study was to explore specific processes of inhibition that result in age-related OTV. Additionally, evidence supporting the inhibition deficit theory typically has been based on correlative studies; few experimental studies have directly investigated the role of inhibition in OTV.

In summary, we aimed to examine the role of different inhibition functions on the age-related increase in OTV. Experiment 1 investigated whether age-related OTV is associated with the pre-mechanism of inhibition (access function), while Experiment 2 investigated whether age-related OTV is related to a post-mechanism (deletion or restraint function).

## Experiment 1

The aim of this experiment was to investigate whether age-related OTV is related to pre-mechanism (access) deficits of inhibition in elderly adults. The access function refers to the prevention of irrelevant information from accessing working memory or the center of attention; therefore, a task designed to investigate the access function should select material that has not entered working memory or formed linkages in the brain. The referential communication task was used to assess old adults’ OTV or conversation (Hupet et al., 1993; Arbuckle et al., 2000). In this task, subject and experimenter were assigned identical sets of stimuli that were arranged in two different sequences, and the subject was required to determine the ordering of the experimenter’s stimuli based on the experimenter’s description and rearrange his/her stimuli sequence to match that of the experimenter’s. The stimuli are usually abstract pictures with no commonly accepted labels. Thus, the subjects’ own knowledge and experience are somewhat irrelevant; instead, each subject must focus on finding references that are meaningful to his or her particular matcher. When describing unfamiliar abstract figures, the subject must come up with a description that the experimenter is able to relate to; thus it must be generic (e.g., “Looks like a kid running left”), not specific to the subject’s own experience (e.g., “Looks like my neighbor kid”). Based on this paradigm, the referential communication task is considered to be not a self-involved communication task, which may not give subjects irrelevant information linkage with their inner side experience which exists in their memory.

If age-related OTV is associated with the pre-mechanism of inhibition, the elderly adults with high-level OTV should experience more interference from the outside irrelevant stimuli. To test this hypothesis, we embedded irrelevant stimuli into the current referential communication task so that they were introduced before the language production task. In the referential communication task, the interference condition variable included interfered and non-interfered levels. In the interfered condition, participants were asked to ignore interfering stimuli that were similar to the target stimuli while they tried to rearrange target stimuli.

### Methods

#### Participants

Fifty-nine elderly adults and 62 young adults were randomly recruited. Thirty-six of the elderly adults were women and 23 were men (*M*_age_ = 67.00 years, *SD* = 6.33, range: 60–90 years). Thirty-eight of the young adults were women and 24 were men (*M*_age_ = 22.31 years, *SD* = 2.47, range: 18–28 years). The first language of all participants was Chinese, and all participants’ vision and hearing were normal.

The baseline levels of OTV were assessed by five widely known public events of Chinese society. According to previous research (James et al., 1998), individual would produce more OTV on autobiographical topic than on non-autobiographical topic. To avoid overestimating the OTV level, this study adopted public events with less self-involvement to assess participants’ baseline OTV level. The participants were asked to talk about their opinions on each event without time limitation. The baseline item verbosity was 0.75 ± 0.30 for the old adults, and 0.57 ± 0.43 for the young adults. The baseline extent OTV was 3.46 ± 2.13 for the old adults, and 2.55 ± 2.23 for the young adults. See the Outline of the structured interview on public events in Appendix.

#### Design

The design was a 2 (interference condition: interfered/non-interfered) × 2 (age: young/old) mixed design, in which the interference condition was a within-subjects variable and age was a between-subjects variable. The dependent variables were item verbosity and extent of OTV.

#### Materials

##### The referential communication task

The stimuli in the referential communication task (Hupet et al., 1993; Arbuckle et al., 2000) were abstract pictures composed of tangram puzzles that did not have commonly accepted labels (e.g., “triangle” or “square”). In this task, the participant and experimenter were given two identical sets of stimuli in two different sequences. They were separated by a screen that prevented them from seeing one another’s arrays and gestures. In the referential communication task, the participants’ own knowledge and experience were somewhat irrelevant. Instead, each participant was expected to focus on finding the references that were meaningful to his or her particular matcher (experimenter). When describing unfamiliar abstract figures, the participant was required to provide a description that the experimenter was able to understand.

**FIGURE 1:**
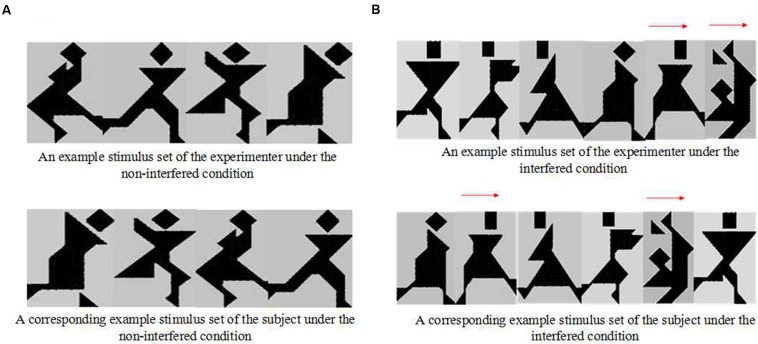
The materials for (A) the non-interfered condition and (B) the interfered condition in Experiment 1.

In the non-interfered condition (examples showed as **Figure 1A**), participants were required to rearrange the four pictures in front of them to form a sequence matching the experimenter’s sequence, based on feedback from the experimenter. In the interfered condition (examples showed as **Figure 1B**), six identical pictures were presented to the participant and the experimenter, two of which were interference pictures (marked by wooden sticks). The participant was asked to ignore the two interference pictures and only rearrange the other four target pictures. All of the target and interference pictures were obtained from the picture pool randomly.

#### Measurements

Off-topic verbosity has been found to be correlated with vocabulary comprehension abilities, the extroversion–introversion continuum, and physical health (Arbuckle and Gold, 1993; Gold and Arbuckle, 1995). Thus, we used the Extroversion–Introversion Test from the Eysenck Personality Questionnaire (EPQ, Cheng, 1983), and the Vocabulary Subtest of the Wechsler Adult Intelligence Scale (WAIS-V, Gong, 1992) to investigate these relationships in the current sample. Clock drawing test (Tuokko et al., 1992) was used to screen participants without Alzheimer disease. Demographic variables and the health status of participants also were recorded.

#### Coding and Rating of OTV

Based on the widely used coding system of OTV (Arbuckle and Gold, 1993; Pushkar et al., 2000) and on Pushkar’s suggested methods (Pushkar et al., 2000), we conducted many rounds of coding and discussion to eventually develop a coding system that could be applied to the Chinese culture.

We coded the OTV of the participants’ answers on two indicators: item verbosity and the extent of OTV.

##### Item verbosity

The item verbosity for each item was scored as 0 or 1; answers that contained any extraneous material were regarded as instances of OTV, and the corresponding item was scored as a 1. Answers that contained no irrelevant information were scored as 0. Item verbosity was defined as the number of answers on which the subjects provided off-topic material divided by the total number of questions. For instance, if a participant’s answers contained extraneous material in three of five questions in baseline level of OTV assessment, his/her item verbosity score was 0.6.

##### Extent of OTV

Responses that contained irrelevant material were scored for the extent of irrelevant content, and the maximum possible score was 9. Next, the extent of OTV scores were calculated as the total of extent ratings for all answers divided by the total number of questions asked. The specific operational definitions were as follows: one point was given when the content of the speech was predominantly related to the current topic, but 1–2 sentences deviated from the topic (or 5–10% of the content was off-topic); three points were given when the topics of the speech that might have large backgrounds, the speech exhibited some contacts with those topics, some of the topics of the speech deviated from the current topic, and approximately 30% of the speech was off-target; five points were given when the topics of the speech might have large backgrounds, the speech exhibited some contacts with those topics, some of the topics of the speech deviated from the current topic, the number of off-target topics (i.e., topic not related to the central theme) was no less than 1, and approximately 50% of the speech was off-target; Seven points were given when the topics of the speech did not connect well with the given topic, the topics of the speech deviated from the current topic, the number of off-target topics was no less than 2, and approximately 70% space of the speech was off-target; Nine points were given when the topics of the speech were not closely connected to the given topic, the topics of the speech deviated from the current topic, the number of off-target topics was no less than 3, and approximately 90% of the speech was off-target.

##### Examples of verbosity transcripts and scoring

We illustrated the scoring with examples in the description of the first stimuli in **Figure 1B**. In Experiment 1, the highest score on extent OTV was 5 among all the participants, so we only gave examples of extent OTV from score “0” to score “5”.

(1) Item = 0, Extent = 0: “Is the top part of the first one shows an angle of the square”;
(2) Item = 1, Extent = 1: “The first one looks like a woman, the top part of it shows a right angle of a square ”;
(3) Item = 1, Extent = 3: “Is the top part of the first one shows an angle of the square? It looks like a woman who is walking with her head down. She is very shy, and...her waist is a little thick. She may be in a bad mood. ”;
(4) Item = 1, Extent = 5: “The first one looks like a shy woman who is walking with her head down. And...it seems that she is wearing an apron. Maybe she is___. She held her hands against her heart. She walked with long steps. By the way, I have ever been to ___ and worked on a foreign aid project decades ago.”

The raters were three college students who majored in psychology. Prior to the present study, a preliminary study was conducted to develop an OTV coding system that was based on the content of speech observed in the preliminary experiment. Three raters rated all the responses in the preliminary study, and the inter-rater reliability was calculated with data from the preliminary study. The inter-rater reliability was acceptable (0.89) and the consistency coefficient for item verbosity with the extent of OTV was 0.86. In the final experiment, each student rate done third of the content of the speech.

#### Procedure

Background information, including age, gender, and years of education was collected after the participants entered the laboratory. The participants were asked to rate their own health status, with 1 = excellent health, 2 = good health, and 3 = poorer health. The EPQ-E (Cheng, 1983) and the WAIS-V (Gong, 1992) were administered to the participants separately. Then, each of the participants completed six trials (three interfered and three non-interfered condition trials) of the referential communication task. The elderly participants took an average of 45 min to complete this task, and the younger participants took an average of 30 min.

This study was approved by the Ethics Committee of School of Psychology, Beijing Normal University and written informed consent was obtained from all participants.

#### Data Analysis

A professional transcription company transcribed the audiotapes. The transcripts of each participant were given identification numbers and then randomly assigned to one of the three scorers. Next, the scorers coded the participants’ item verbosity and extent of OTV. Group differences in demographic variables, the EPQ-E and the WAIS-V were examined by using independent *t*-test or a chi-square analysis. Pearson’s correlation analysis was used to examine the relationships between baseline OTV and other variables, including demographic variables, the EPQ-E and the WIR-V. Two-way Analysis of variance (ANOVA) was conducted on the item verbosity and the extent of OTV to compare OTV between two age groups. Simple effect analysis was further performed to evaluate the effect of interference condition in each age group.

### Results and Discussion

Significant differences between the two age groups were found in terms of health status, with older adults having worse health (*p* < 0.05). No differences between age groups were found for gender, education, or scores on the EPQ-E and the WAIS-V. The background of participants are shown in **Table 1**.

**Table 1:** Demographic information and baseline performances of the participants in participants recruitment stage.

	Young group (*N* = 62)	Old group (*N* = 59)	*t*
Age	22.31 ± 2.47	67.00 ± 6.33	–51.64^∗∗∗^
Gender (Male/Female)	24/38	23/36	0.03
Years of education	15.15 ± 1.99	14.39 ± 2.43	1.88
Health status	1.66 ± 0.65	2.32 ± 0.66	5.56^∗∗∗^
Clock drawing scores	3.23 ± 0.79	3.64 ± 0.59	–1.31
EPQ-E	13.26 ± 5.01	14.32 ± 3.45	1.37
Word comprehension	15.26 ± 2.75	14.63 ± 3.19	1.17
Baseline item verbosity	0.57 ± 0.43	0.75 ± 0.30	–2.53^∗^
Baseline extent off-topic verbosity (OTV)	2.55 ± 2.23	3.46 ± 2.13	–2.29^∗^

*In this and subsequent tables, ^∗∗∗^indicates statistical significance at the 0.001 level and ^∗^indicates statistical significance at the 0.05 level.*

Correlation analysis was performed between baseline OTV and age, education, health status, personality and vocabulary ability. Results showed that the baseline of item verbosity (*r* = 0.25, *p* = 0.005) and the extent of OTV (*r* = 0.24, *p* = 0.007) was only significantly related to age, with older adults being correlated with an increased incidence of OTV. Thus, education, health status, personality and vocabulary ability were not controlled in following analysis.

**Table 2** shows the descriptive statistics for item verbosity and extent of OTV in the referential communication task.

**Table 2:** Descriptive statistics for OTV in the referential communication task from Experiment 1 (*M* ± *SD*).

	Young group *(N =* 62*)*		Old group *(N =* 59*)*	
	Item verbosity (Iv)	Extent OTV (Ev)	Item verbosity (Iv)	Extent OTV (Ev)
Interfered	0.67 ± 0.42	1.46 ± 1.15	0.97 ± 0.14	2.05 ± 1.13
Non-interfered	0.66 ± 0.43	1.22 ± 1.05	0.98 ± 0.13	2.04 ± 1.08

A 2 (age: young/old) × 2 (interference condition: interfered/non-interfered) two-way ANOVA was conducted on item verbosity. The results revealed a main effect of age, *F*(1,117) = 28.98, η^2^ = 0.20, *p* < 0.01, which showed that the item verbosity scores of the elderly adults were much greater than were those of the young adults. However, no interaction was found between age and interference condition on item verbosity, *F*(1, 117) = 0.655, η^2^ = 0.01, *p* > 0.05.

**FIGURE 2:**
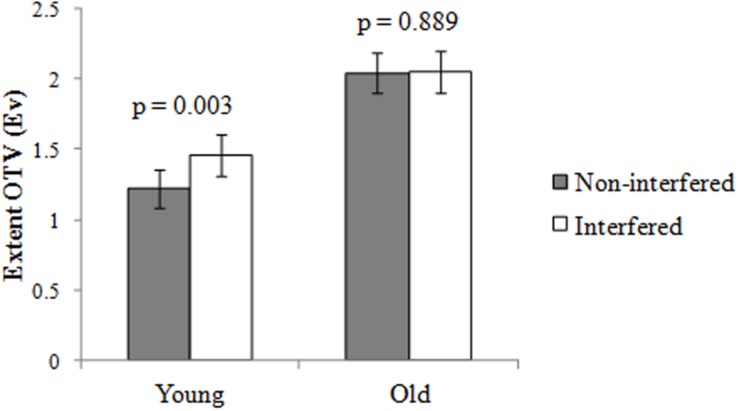
**The role of access function in the age-related off-topic verbosity (OTV): The interaction between age and interference condition on the extent of OTV in Experiment 1.** It shows the mean extent of OTV scores for the individuals. Error bars represent standard errors of the mean.

A 2 (age: young/old) × 2 (interference condition: interfered/non-interfered) two-way ANOVA was conducted to examine the age differences on the extent of OTV. Results revealed a main effect of age, with the elderly adults exhibiting a greater extent of OTV than the young adults, *F*(1,116) = 11.18, η^2^ = 0.09, *p* < 0.01. There was an interaction between age and interference condition, *F*(1,116) = 4.08, η^2^ = 0.03, *p* < 0.05. Simple effects (**Figure 2**) analyses revealed that the extent of OTV was greater in the interfered condition than in the non-interfered condition for the young group, *F*(1,117) = 9.09, *p* < 0.01, but no difference between the two conditions was found for the elderly group, *F*(1,117) = 0.02, *p* > 0.05.

The results revealed no difference between the two levels of interference conditions for the elderly participants, indicating that the OTV of the elderly might not correlate with the pre-mechanism of inhibition. The OTV of younger adults was greater in the interfered condition than in the non-interfered condition. This finding suggested that young adults who exhibited OTV might have a deficit in the access function that may not decline with age.

## Experiment 2

The aim of Experiment 2was to examine whether age-related OTV is caused by deficits in the post-mechanism of inhibition. As mentioned before, post-mechanisms which include deletion and restraint functions mainly exert their effects after interfering information is activated. To explore the effects of deletion and restraint functions on OTV, we designed a task paradigm which can generate previously relevant but currently irrelevant information or induce strong but inappropriate information from participants’ inner experiences. Based on this purpose, a self-involved event interview task was adapted in this experiment. In this task, three questions about different experiences in one’s life, e.g., pertaining to education, friendship or teacher experiences, were presented to the participants, who were instructed to think carefully about their answers to each question (to ensure activation of content related to all three questions). Then, they were asked to answer only one of the questions. Participants did not know which question they would be asked to answer when they thought about the answers. Thus, the activated information related to the other two questions was irrelevant for the question requiring an answer. Additionally, these self-involved questions were also related to participants’ own knowledge and experience, which may induce their strong but inappropriate responses. Thus, according to their answers, we can detect whether participants’ OTV were related to the delete or restraint function.

To complete the task successfully, participants had to delete the content that was no longer relevant from their working memory. In this task, the item homogeneity variable was manipulated by including homogeneous and heterogeneous conditions. In the homogeneous condition, the three questions were related to the same topic (e.g., education), while in the heterogeneous condition, the questions were all unrelated. We hypothesized that the homogenous condition would activate more irrelevant information than would the heterogeneous condition. Thus, the homogenous condition would require a greater ability to delete irrelevant information. If age-related OTV were correlated with the deletion function of inhibition, more OTV would be expected in the homogeneous condition.

### Methods

#### Participants

Participants were the same as in the experiment 1.

#### Design

The design was a 2 (item homogeneity: homogeneous/ heterogeneous) × 2 (age: young/old) mixed design, with item homogeneity as a within-subjects variable and age as a between-subjects variable. The dependent variables were item verbosity and extent of OTV.

Homogeneity was a within-subjects variable, but we could not ask the participants to answer the same questions (under different conditions) twice. Therefore, we used a between-item, within-subject design. The participants in each age group were divided into subgroups A and B, and each participant completed six trials three in the homogeneous and three in the heterogeneous conditions). In each corresponding trial, the questions that were answered by the participants of subgroup A in the heterogeneous condition were the same as the questions that were answered by the participants of subgroup B in the homogeneous condition. For example, in trial 1, the questions presented to the participants of subgroup A were three heterogeneous questions, and those presented to the participants of subgroup B were the same questions as those presented in the homogeneous conditions. The questions answered by participants of subgroups A and B were the same (e.g., “Please talk about the most successful thing you have ever done.” See **Table 3**).

**Table 3:** Between-item and within-subject design in experiment 2.

	Participants from Subgroup A	Participants from Subgroup B
Trial 1	**Heterogeneous condition**	**Homogeneous condition**
	Item 1: Please talk about the celebrity you most revere;	Item 1 Please talk about the place you admire most about yourself;
	Item 2: What will happen if the opinions of your children differ from yours? Give an example;	*Item 2 Please talk about the most successful thing you have ever done;*
	*Item 3: Please talk about the most successful thing you have ever done.*	Item 3 please talk about the greatest achievement you have achieved in the eyes of others.
Trial 2	**Homogeneous condition**	**Heterogeneous condition**
	*Item 1: Please talk about your most memorable journey.*	Item 1: Please briefly introduce your educational background.
	Item 2: Please talk about your favorite scenic spot.	*Item 2: Please talk about your most memorable journey.*
	Item 3: Whom would you most like to travel with?	Item 3 Please briefly introduce your advantages and disadvantages.
……		
Trial 6	**Homogeneous condition**	**Heterogeneous condition**

*The items in italics were the target questions that participants were required to answer.*

#### Material

##### The self-involved event interview

In this task, participants were presented with three self-related questions and asked to think carefully about each of the questions for 1 min. Every question was presented after confirming that the participants knew how to answer them. After all three of the questions were presented and the participants indicated that they knew how to answer each of them, the experimenter selected one of the questions and asked participants to answer it. Thus, the content activated by the other two questions was previously, but not currently, related to the task. The designated questions to be answered were presented in random order to eliminate the effect of participants’ expectations.

This task included homogeneous and heterogeneous conditions. Fifteen undergraduates, who were non-psychology majors, were invited to rate the homogeneity of the three questions presented in each trial on a 5-point scale (5 = high homogeneity to 1 = low homogeneity). Homogeneity was defined as the similarity of the content activated by different questions. The content activated by homogeneous questions was similar. Questions that scored 1 or 2 points were used as heterogeneous topics, and those that scored higher than 3 points were used as homogeneous topics. First, we calculated the average homogeneity scores for each series of topics (by calculating the arithmetic mean values of the pair-wise correlations among each set of topics), then we calculated the average scores of the homogeneous and heterogeneous conditions. The average score of the heterogeneous topics was 1.63 points, and the average score of the homogeneous topics was 3.95.

To evaluate the validity of the self-involved interview task, three psychological experts were invited to rate the extent of this task whether reflect the definitions of deletion and restraint functions from 1 (reflect the definitions entirely) to 5 (not reflect the definitions entirely). All of the experts rate 1.

#### Coding and Rating of the OTV

##### Coding and rating

The coding rules that were used were identical to those of Experiment 1. Moreover, we coded the content of the OTV as reflected by the deletion and restraint functions. If the content of the OTV was related to the other two questions, then the extent of OTV was rated as reflecting the deletion function, and if the content of the OTV was self-related and not correlated with any of the three questions that were presented to the participants, the extent of OTV was rated as reflecting the restraint function^1^. For instance, when the participant was asked to “talk about the most successful thing you have ever done” in the heterogeneous condition in trial 1: if the participant told the experimenter about what will happen if the opinions of his/her children differ from his/hers, then this content was rated as reflecting the deletion function; if the participant told the experimenter about his/her unhappy marriage life, which was not correlated with any of the three questions, then this content was rated as reflecting the restraint function. DEv represents the extent of OTV that reflects the deletion function, and REv represents the extent OTV that reflects the restraint function. Both DEv and REv were included in rater reliability calculation as mentioned in Experiment 1.

##### Examples of verbosity transcripts and scoring

We illustrated the scoring with examples in the heterogeneous condition of Trial 1 in Experiment 2.

*Question:* “Please talk about the most successful thing you have ever done.”

Answers:

(1) Item = 0, Extent = 0: “I did a good job with my daughter and she is outstanding”;
(2) Item = 1, Extent = 1: “There was no successful thing that I have ever done. Having say that, I think my most successful thing, is that I have two obedient children. However, my marriage life is unhappy”;
(3) Item = 1, Extent = 3: “That I think my most successful thing, uh, is when I was the leader of the propaganda department, I often provided the valuable news report. Therefore, I got very positive evaluation from the leadership and the staff of the company. Because most of my time was spent on my work, I didn’t take care of my children. They began to alienate me, and they didn’t listen to me. Then, they usually had conflict with me. Now it seems that the loss outweighs the gain”;
(4) Item = 1, Extent = 5: “Well, my son is very filial. When he is a little child, I always worked until very late. At that time, he was very naughty, and I used to believe he was a bad child. We always held conflicting opinions. However, I learned how to get along with children from books afterward. Then, he became very sensible. Uh, two generations have different ways of thinking, we should learn to replace thinking. Maybe you don’t know what we generation think. My son is bigger than you, he is 33. Our idea is different, a lot of things is not the same. We generation suffered from___, while you didn’t experience real hunger. We are different. We should look forward, as opposed to looking backward, and we couldn’t always recall the past”;
(5) Item = 1, Extent = 7: “I have never considered the most successful things in my life. Having say that, the most successful thing, is that I didn’t forget I was born in a revolutionary family, I always support the leadership of the party, to share the joys and sorrows in my 40 years career life. I have worked for nearly 40 years, I have experienced so many___ in these 40 years…… (omitted here one thousand characters about how she worked in these years)……I really admire and respect my excellent leader…… (omitted here one thousand characters about her leader and the trivial they have together experienced)……Now, I have been retired and I can arrange my own life well. All my friends and neighbors like me very much…… (omitted here about one thousand characters about her advantages)……”
(6) Item = 1, Extent = 9: “My children all make good living now. I say. I have three children, the first and the second were sent down to the countryside, and the educated youth went to the countryside became the city’s fashion in that era. The second one is a girl, who was sent to the countryside. The eldest one was a worker and he worked at oil refinery. The youngest one, belonged to the right generation as he could participant in the college entrance examinations. He could take the exam after his graduation from high school. However, he was not good at science and engineering, and he was willing to work. At that time, our organization had a plenty of unloading car, what ah, he was willing to work. I criticized him, ‘your brother and sister could hardly wait for the college entrance examination, they had no opportunity, But, you have.’ However, it did not help the matter in spite of my long time preaching, he was still willing to work, I have thought a lot of ways to persuade him afterward. However, this child was special introverted, and he didn’t love to talk, engineering almost, almost in science and engineering. He felt his engineering was not so good, and his goal was to work, have to work. In fact, we were not the family that couldn’t afford their children’s tuition. However, he was not obedient, usually had conflict with me……(omitted here 2000 characters about how she left nothing untried to put the youngest child on the right track)……Now, he was a national civil servant and I think I am successful, He has lived with me now, after all, he can’t afford to buy a house. I and my husband were graduated from Shanxi University, Taiyuan. He studied engineering and I studied teaching. After graduation he was assigned to the ministry of railways, and I was assigned to the Chaoyang Education Bureau……(omitted here one thousand characters about her working life)……Well, I say, my three children are pretty good, all have good jobs, I think, it is successful to give the child a good living. Do you think so?”

#### Data Analysis

Two-way ANOVA was conducted to compare age differences on the item OTV, the DEv, and the REv. Simple effect analysis was further performed to evaluate the effect of homogeneity condition in each age group.

### Results and Discussion

**Table 4** shows the descriptive statistics for item verbosity and extent of OTV in this task.

**Table 4:** Descriptive OTV statistics in the self-involved event interview task from Experiment 2 (M ± SD).

	Young group (*N =* 62)			Old group (*N =* 59)		
	Item verbosity (Iv)	Extent OTV (DEv)	Extent OTV (REv)	Item verbosity (Iv)	Extent OTV (DEv)	Extent OTV (REv)
Heterogeneous	0.70 ± 0.41	0.65 ± 0.98	2.36 ± 1.96	0.97 ± 0.18	0.29 ± 0.65	3.64 ± 2.19
Homogeneous	0.69 ± 0.98	1.06 ± 1.43	2.15 ± 1.93	0.99 ± 0.06	1.70 ± 1.74	4.18 ± 2.45

A 2 (age: young/old) × 2 (homogeneity condition: homo geneous/heterogeneous) two-way ANOVA conducted on item verbosity showed a main effect of age, *F*(1,118) = 27.42, η^2^ = 0.19, *p* < 0.001. The scores for item verbosity of the elderly adults were much higher than were those of the young adults. However, there was no interaction between age and homogeneity condition, *F*(1,118) = 1.02, η^2^ = 0.01, *p* > 0.05.

**FIGURE 3:**
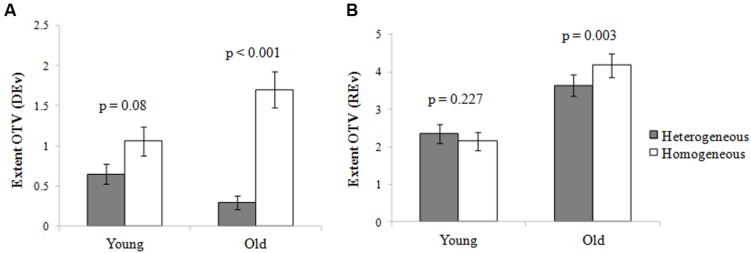
**The role of deletion and restraint functions in the age-related OTV: The interaction between age and homogeneity in the extent of OTV (A) that reflected the deletion function (DEv) and (B) that reflected the restraint function (REv) in Experiment 2.** It shows the mean extent of OTV scores for the individuals. Error bars represent standard errors of the mean.

A 2 (age: young/old) × 2 (homogeneity condition: homo geneous/heterogeneous) two-way ANOVA was conducted to examine age differences in the DEv. The results showed that, for DEv, there was a significant interaction between age and homogeneity, *F*(1,117) = 7.16, η^2^ = 0.06, *p* < 0.01. Simple effects analyses (**Figure 3A**) showed that for the elderly adults, DEv in the homogeneous condition was greater than that in the heterogeneous condition, *F*(1,118) = 33.80, *p* < 0.001. In the young adults, the DEv in the homogeneous condition was not significantly higher than that in the heterogeneous condition, *F*(1,118) = 3.12, *p* < 0.05.

A 2 (age: young/old) × 2 (homogeneity condition: homo geneous/heterogeneous) two-way ANOVA was performed to examine age differences in the REv. The results showed that for the extent of OTV reflecting the function of restraint (REv), there was a significant main effect of age, *F*(1,117) = 17.36, η^2^ = 0.13, *p* < 0.001, and a significant interaction between age and homogeneity, *F*(1,117) = 7.46, η^2^ = 0.06, *p* < 0.01. Simple effects analyses (**Figure 3B**) showed that, for the elderly adults, the REv in the homogeneous condition was greater than that in the heterogeneous condition, *F*(1,118) = 9.17, *p* < 0.01; for the young adults, no significant differences In REv were found between the two conditions, *F*(1,118) = 1.48, *p* > 0.05.

From the analysis of the DEv and that of the REv, we observed that the OTV in the homogeneous condition was higher than was that in the heterogeneous condition for older adults, but not for young adults. Therefore, it is thought that age-related OTV is closely related to the post-mechanism of inhibition (including the deletion and restraint functions).

## General Discussion

Deficits in inhibition likely lead to age-related OTV and affect all aspects of language production in elderly adults, including non-autobiographical topics (i.e., the referential communication task of Experiment 1) and autobiographical topics (i.e., the self-involved interview task of Experiment 2) (Arbuckle and Gold, 1993; Gold and Arbuckle, 1995; Pushkar et al., 2000; Wills et al., 2012). Although the inhibition deficit theory may explain the occurrence of OTV during the discussion of general topics, the theory and its supporting evidence do not clearly explain the mechanism of inhibition in age-related OTV. The results of the present study showed that the OTV produced by older adults was closely related to the post-mechanism of inhibition, while OTV produced by young people was likely related to non-age-related deficits in the access function of inhibition. As reported in Experiments 1 and 2, no differences were found in item verbosity, so the following discussion is centered on the influence of age differences on the extent of verbosity.

### The Role of the Pre-mechanism of Inhibition in Age-related OTV

The results of Experiment 1 found that the OTV of elderly adults might not correlate with the pre-mechanism of inhibition. This finding may have resulted from two reasons. One reason is that the ability to prevent irrelevant information from entering working memory may not decline in the elderly (Dumas and Hartman, 2008; Feyereisen and Charlot, 2008). Feyereisen and Charlot (2008) asked participants to complete tasks reflecting the access function (including problem solving and a distracted reading task); the participants were required to select target information upon simultaneous presentation of irrelevant information. No significant age differences were found in tasks that reflected the access function, which indicates that the access function might not decline with age. However, there was no direct evidence about unimpaired access function of old adults in present study The other reason may be that the interference condition in Experiment 1 was relative less cognitive demanding. Research showed that the performances of some inhibition tasks, like negative priming, in old adults did not decrease if resisting the interference needed less cognitive control (Guerreiro et al., 2010). In our study, if the interference become strong, like interference location is very close, even overlaps partly, to target stimuli, the interference effect may be relative obvious Therefore, whether the access function is associated with age-related OTV still need further experimental evidence.

We were surprised to find that the young participants exhibited more OTV under the interfered condition than under the non-interfered condition. This finding suggests that the OTV of young adults might be related to deficits in the pre-mechanism of inhibition. If the OTV of young adults does relate to the pre-mechanism of inhibition, then interfering stimuli may need to be reduced when talking with young adults with high levels OTV. Less irrelevant information may improve young adults’ quality of speech production and efficiency of communication. Whereas, the surprising finding of younger adults showing more OTV in the context of irrelevant items could be perhaps a cohort effect. Young adults live in a environment where there are more frequent demands to disengage from one’s current tasks – demands placed on the young through social media, various software, and smartphone alerts etc. Older adults, by contrast, are less exposed to this environment. Thus, the access function may play more important role in young adults’ OTV than in old adults’ OTV.

### The Role of the Post-mechanism of Inhibition on Age-related OTV

The results of Experiment 2 revealed that age-related OTV might be closely related to the post-mechanism of inhibition. Compared to the young adults, the elderly adults with high OTV were not able to delete information that was previously relevant and currently irrelevant to the task. Therefore, they exhibited more OTV, and the content of their OTV was closely related to the information that was activated in the previous task. Previous studies have found that the deletion function significantly declined in elderly adults (Dumas and Hartman, 2008; Feyereisen and Charlot, 2008). Feyereisen and Charlot (2008) asked participants to complete a task involving the deletion function. The source of the interference was the stimulus information from a previous task that was irrelevant to the current one; the task did not come from the environment. Their results showed that elderly people exhibited poorer performance than did young adults when they were required to delete information that was no longer relevant. Dumas and Hartman (2008) reported similar results that the age-related decline in the deletion function affected a wide range of cognitive functions.

Compared to the young adults, the elderly adults were also unable to restrain the activation of some advantageous infor mation and, therefore, appeared to have more OTV. The content of their speech was closely related to the impressive amount of autobiographical information they have. Age-related OTV was accompanied by age-related declines in restraint function. This finding suggests that OTV in older adults was related not only to the previous topic, but also to the self-initiated information of their autobiographical memories. Thus, their OTV may have been associated with their declining restraint function or their rich life experiences. Greater amounts of self-related personal information resulted in more irrelevant information in their OTVs. Previous studies have revealed that the restraint function of inhibition generally declines in older adults (Dumas and Hartman, 2008; Feyereisen and Charlot, 2008); on the basis of this finding, a wealth of life experience might lead to more OTV.

In sum, age-related OTV might be not related to the pre-mechanism of inhibition, but was associated with age-related declines in the post-mechanism of inhibition. Although we found that age-related OTV was closely related to the post-mechanism of inhibition, it remains unclear whether the deletion or restraint function plays a larger role in age-related OTV. The scores of DEv were lower than that of REv for both old and young group in Experiment 2. According to these results, we may infer that restraint function play a relative important role in age-related OTV. The results of Arbuckle and Gold (1993) also showed that verbosity was significantly related to performance on the TMT (restraint function) rather than performance on the WCST (deletion function). However, the present study could not directly explain which function of the post-mechanism of inhibition play a more important role on OTV. Further experimental evidence is needed to clarify this question.

### Limitations and Future Directions

This study attempted to clarify the mechanism by which age-related declines in inhibition might lead older adults to exhibit OTV. It also examined whether and how deficits in inhibitory function might lead to OTV in younger adults. These areas deserve closer investigation and have the potential to contribute to a better understanding of the relationship between changes in cognitive function and older adults’ communicative behavior and abilities. Currently, little research has been conducted to investigate the relationship between age-related OTV and the inhibition function. This research was designed to fill gaps in the existing knowledge about the potential relationship between declining inhibitory function and age-related OTV. The findings of this study warrant further examination.

Nevertheless, the present study still had some limitations. First, the scores of extent of OTV in Experiment 1 were generally lower than those scores in Experiment 2. Is this phenomena related to the tasks features (non-autobiogra phical/autobiographical)? The reasons of old adults exhibited higher OTV in autobiographical topics are needed to be deeply considered. Second, although we found that age-related OTV was closely related to the post-mechanism of inhibition, it remains unclear whether the deletion or restraint function plays a larger role in age-related OTV. The scores of DEv were lower than that of REv for both old and young group in Experiment 2. According to these results, we may infer that restraint function play a relative important role in age-related OTV. However, further experimental evidence is needed. Third, we test the “access” function in a different domain (describing abstract images) in contrast to “deletion” and “restraint” functions, which we test under more natural verbal conditions. The absence of a deficit in “access” function might be partly related to the more unusual domain to be tested. Thus, we need to be more careful drawing conclusions from these results. Fourth, though we designed two experiment tasks to reflect three inhibition functions, we did not use measurement tasks about inhibition and analyze the correlation between inhibition performance and the OTV in current study. It should be considered in further study. Fifth, this study attempted to explain the mechanisms of inhibition in age-related OTV. However, OTV is a complex process involving the combined effects of cognitive and social factors that cannot be explained entirely by inhibitory functions. It may be wise to combine different theories to explain age-related OTV.

## Author Contributions

SY and HP conceived the study design. SY participated in the data collection, performed the statistical analysis, and drafted the manuscript. HP is the principal investigator of this project, and supervised the statistical analysis and the manuscript writing and revision. All authors read and approved the final manuscript.

## Conflict of Interest Statement

The authors declare that the research was conducted in the absence of any commercial or financial relationships that could be construed as a potential conflict of interest.

## Footnotes

1 Note: this study did not consider the restraint function when coding the extent of OTV in Experiment 1 because the OTV content that reflected the restraint function accounted for less than 10% of the total content.
