# Supplementary material for: The Role of Inhibition in Age-related Off-Topic Verbosity: Not Access but Deletion and Restraint Functions
Source: Front Psychol. 2016 Apr 26;7:544. doi: 10.3389/fpsyg.2016.00544 (PMC4844921; doi:10.3389/fpsyg.2016.00544)
Supplement: Supplementary file 1 [file Data_Sheet_1.DOCX]

**Appendix**

**Interview outline on public events in China**

1. During October 1-5 of 2010, the 41st World Expo was held in Shanghai of China.Would you please briefly talk about its influence on the life of Shanghai citizens.

2. Please briefly talk about your opinion on people’s reaction to “5.12 ”Wenchuan Earthquake.

3. Who do you think should be blamed most in the issue of “Little Yueyue”?

4. Please briefly talk about your opinion on the effect of“2008 Beijing Olympic Games” on China's economy?

5. Please briefly talk about your view on current retiree health care system.
